# Comparison of the Efficacy of Kligman's Formula Combined With 30% Topical Metformin Versus Kligman's Formula Alone in the Treatment of Melasma

**DOI:** 10.1111/jocd.70983

**Published:** 2026-06-12

**Authors:** Fariba Iraji, Fatemeh Mokhtari, Zeinab Kassab, Sarah Seyedyousefi, Jaleh Varshosaz

**Affiliations:** ^1^ Dermatology, Faculty of Medicine Isfahan University of Medical Sciences Isfahan Iran; ^2^ Skin Diseases and Leishmaniasis Research Center Isfahan University of Medical Sciences Isfahan Iran; ^3^ Pharmacognosy, School of Pharmacy and Pharmaceutical Sciences Isfahan University of Medical Sciences Isfahan Iran

**Keywords:** melasma, metformin, triple combination creams

## Abstract

**Background:**

Melasma is a common hyperpigmentation disorder primarily affecting women, influenced by ultraviolet exposure, hormones, and genetics. Kligman's formula is a first‐line therapy, but emerging evidence suggests that topical metformin, an antidiabetic with antimelanogenic effects, may enhance treatment outcomes. The objective of this study is to compare the efficacy and safety of Kligman's formula with and without 30% topical metformin in bilateral facial melasma.

**Methods:**

In this randomized, double‐blind, split‐face clinical trial, 31 patients with symmetrical facial melasma applied Cream A (Kligman's formula + 30% metformin) to one side and Cream B (Kligman's formula alone) to the other for 2 months. Outcomes included MASI scores, repigmentation grading, patient satisfaction via Visual Analog Scale (VAS), and side effect profiles assessed at baseline, post‐treatment, and 2 months after discontinuation.

**Results:**

Both treatments significantly reduced pigmentation and improved homogeneity. Cream B showed a slightly greater reduction in darkness, while Cream A led to more stable repigmentation and fewer cases of post‐inflammatory hyperpigmentation. No significant differences were found between post‐treatment and follow‐up scores. VAS scores indicated high patient satisfaction across both groups.

**Conclusion:**

Adding 30% topical metformin to Kligman's formula is safe and comparably effective in melasma treatment, offering potential benefits in pigment stability and reduced PIH. Although differences were not statistically significant, clinical trends support further investigation through larger, long‐term studies.

**Trial Registration:**

ClinicalTrials.gov identifier: IRCT20150706023081N2

## 1 Introduction

Melasma is a chronic skin condition primarily affecting women and characterized by symmetrical, hyperpigmented patches on sun‐exposed areas, especially the face [1]. It is commonly associated with triggers such as ultraviolet (UV) radiation, hormonal fluctuations, and genetic predisposition [2]. The condition is notably prevalent during pregnancy, impacting an estimated 50%–70% of pregnant women, and is more frequently observed in individuals with darker skin tones, particularly among Asian and Hispanic populations [2, 3]. In Iran, Moin et al. [4] documented a prevalence of 15.8% among women.

Melasma typically manifests in three facial patterns: mandibular, malar, and centrofacial. While its precise pathogenesis remains uncertain, contributing factors include hormonal imbalances, sun exposure, medication use, and genetic susceptibility. Among these, sunlight exposure is widely acknowledged as a key exacerbating factor [5, 6].

Recent histopathological findings suggest that melasma should also be considered a manifestation of photoaging. Changes such as dermal elastosis, angiogenesis, and increased mast cell activity reinforce this perspective. Consequently, melasma is now understood as a persistent dermatologic condition with substantial psychosocial implications, including lowered self‐esteem and emotional distress [7].

Microscopic studies have revealed increased activity and density of melanocytes, along with enhanced melanin production and transfer to keratinocytes and dermal macrophages [8].

Treatments range from topical lightening agents and sunscreens to chemical peels and laser‐based therapies. Among topical agents, hydroquinone is widely accepted as a leading depigmenting agent, typically used either alone or in combination with retinoids and corticosteroids [9]. In a head‐to‐head comparison between 10% topical zinc sulfate and 4% hydroquinone, Iraji et al. [10] found that both agents led to comparable pigment improvement, while zinc sulfate was associated with fewer side effects, making it a promising non‐hydroquinone alternative.

The Kligman formulation, containing hydroquinone, tretinoin, and a corticosteroid, remains a cornerstone of melasma management [11]. Despite its widespread use, variability in its effectiveness has been observed among Asian skin types. Hydroquinone's primary action is the inhibition of melanin synthesis via tyrosinase suppression. However, it also has the potential to cause skin irritation and, in rare cases, ochronosis, prompting regulatory caution in some countries [4].

Other emerging therapies include tranexamic acid (TXA), which interferes with melanogenesis by inhibiting plasmin. Both topical and oral TXA formulations have shown efficacy comparable to hydroquinone but with fewer adverse effects [12].

Furthermore, the FDA‐approved triple combination cream containing hydroquinone, tretinoin, and fluocinolone is highly effective and relatively well‐tolerated. Tretinoin supports epidermal turnover and hydroquinone penetration, while fluocinolone helps mitigate irritation. Numerous studies validate this combination's efficacy, especially in individuals with darker complexions [13, 14].

Metformin, a common antidiabetic agent, has garnered interest for its dermatological applications, including melasma. Evidence suggests that it inhibits melanogenesis through mechanisms involving AMPK activation and mTOR pathway suppression. In vitro and in vivo studies have shown that metformin reduces intracellular cAMP levels and subsequently downregulates MITF and melanogenic enzymes such as tyrosinase, TRP‐1, and TRP‐2 [15].

Topical metformin is often prepared by dissolving the compound in a solvent base of ethanol and propylene glycol. Applied nightly, this formulation has demonstrated promising results in early clinical trials. Studies by Ravikumar et al. and AboAlsoud et al. [16, 17] concluded that 30% topical metformin is both effective and well‐tolerated, offering a potential alternative to traditional regimens.

However, direct clinical comparisons between Kligman's formula with and without topical metformin remain sparse. This study was thus designed to fill that gap using a randomized, split‐face, double‐blind methodology.

## 2 Materials and Methods

Upon receiving informed consent and confirming eligibility, patients were enrolled and asked to complete a checklist documenting demographic data, comorbidities, and potential risk factors.

This 4‐month clinical trial enrolled 31 patients with bilateral facial melasma lesions. A split‐face design was employed, whereby each patient served as their own control.

Our inclusion criteria were adult patients (≥ 18 years) with bilateral symmetrical facial melasma, clinical diagnosis of melasma confirmed by dermatologist, Fitzpatrick skin types III–V, no topical or systemic melasma treatment within 4 weeks prior to enrollment and willingness to use sunscreen and comply with study protocol. The exclusion criteria were as follows: pregnancy or lactation, use of systemic retinoids in the past 6 months, history of hypersensitivity to hydroquinone, tretinoin, corticosteroids, or metformin, active inflammatory dermatoses, infection, or wounds on the face, history of keloid formation, use of photosensitizing medications.

Melasma severity was assessed using the Melasma Area and Severity Index (MASI), which incorporates three variables: the percentage of facial involvement, pigmentation darkness, and homogeneity. The face was divided into four regions: forehead (30%), right malar (30%), left malar (30%), and chin (10%). The modified MASI score was calculated using the formula:

$$\begin{array}{cc} \text{Modified MASI}= & \;0.3\;\left(D+H\right)\;A\;\left(\text{forehead}\right)+0.3\;\left(D+H\right)\;A\;\left(\text{right malar}\right)\\ & \;+0.3\;\left(D+H\right)\;A\;\left(\text{left malar}\right)+0.1\;\left(D+H\right)\;A\;\left(\text{chin}\right) \end{array}$$

Severity grading was based on clinical assessment by a dermatologist, while lesion depth was determined using Wood's lamp examination, which highlights epidermal or dermal melanin deposition. Lesions were classified as epidermal, dermal, or mixed.

Following ethics committee approval and patient consent, individuals with no melasma treatment in the previous month were recruited. A standardized checklist collected medical history, previous treatments, and provided instructions for treatment adherence.

### 2.1 Preparation of Study Creams

- Step 1: Hydroquinone, fluocinolone, and tretinoin powders were finely ground in a mortar. Then, 5 cc of propylene glycol was added and thoroughly mixed.
- Step 2: Separately, 1 g of vitamin C powder and 0.1 g of metabisulfite cream were dissolved in 10 cc of distilled water. Additionally, 30 g of metformin powder was dissolved in 10 cc of distilled water in a separate mortar. These solutions were combined with the initial mixture.
- Step 3: The final mixture was gradually incorporated into the cold cream base and homogenized.

Each patient received two coded creams (A and B), indistinguishable in packaging, texture, smell, and color. Neither patients nor physicians were aware of the cream contents. According to a randomization schedule, patients applied cream A to one side of the face and cream B to the other.

- Cream A contained Kligman's formula + 30% metformin.
- Cream B contained Kligman's formula alone.

The creams were applied once nightly for 2 months, avoiding the periorbital area.

The CONSORT flowchart illustrates the progression of participants throughout the study.

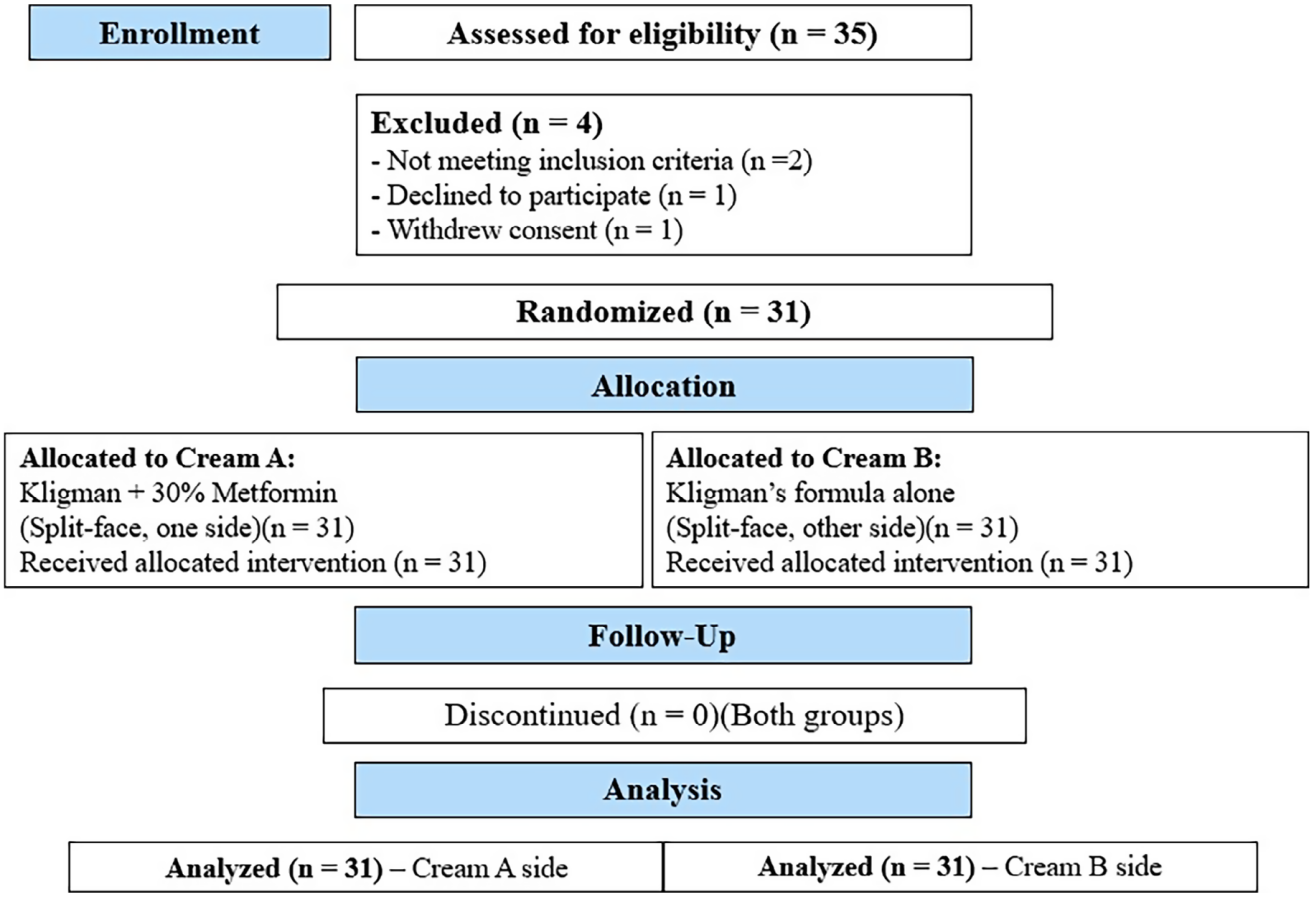

### 2.2 Patient Melasma Assessment

Patients were evaluated at baseline, 2 months after treatment initiation, and 2 months after cessation. Outcomes included MASI score changes, satisfaction measured via Visual Analog Scale (VAS, 0–10), and adverse effects. Data were recorded in a dedicated checklist.

Patients were followed for 2 months post‐treatment to assess relapse, MASI scores, and satisfaction. Standardized photographs of both facial sides were taken at baseline, post‐treatment, and post‐follow‐up.

#### 2.2.1 Grading of Clinical Improvement

Improvement was evaluated based on changes in patch size and pigmentation, categorized as follows:

- G0 (No response)
- G1 (< 25% improvement, Poor)
- G2 (25%–50% improvement, Moderate)
- G3 (50%–75% improvement, Good)
- G4 (> 75% improvement, Excellent)

Photographic assessments were independently reviewed by two dermatologists blinded to treatment allocation.

Sunblock tailored to each patient's skin type was prescribed for daily use. Throughout the study, any adverse effects were documented based on patient interviews and recorded in patient forms.

### 2.3 Statistical Analysis

Descriptive statistics for qualitative variables will be presented using frequency tables and charts. For quantitative variables, central tendency and dispersion indices will be employed. For inferential analysis, the chi‐squared (*χ* ^2^) test will be used for categorical data, and the independent *t*‐test will be applied for comparing means between two groups. Additionally, analysis of covariance (ANCOVA) will be conducted to adjust for potential confounding variables. After data collection and quality control, all data will be entered into a computer and analyzed using SPSS software, version 20.

## 3 Results

### 3.1 Statistical Analysis

The frequency (%) or mean (SD) was used to describe categorical and continuous data, respectively. The unit of analysis for the split‐face design was the treated side. In split‐face analysis, the changes in post‐treatment outcomes from baseline measurements on each side were calculated. Given the small sample size (*n* = 31) and the deviation from normality (indicated by both the Shapiro–Wilk test and kurtosis/skewness statistics), comparisons between the two treated sides over time were performed using a generalized estimating equation (GEE) model, with a Bonferroni correction for multiple comparisons. Categorical variables were compared between the two treated sides using McNemar's test. A *p*‐value of < 0.05 was considered statistically significant. Analysis was performed using SPSS software (version 20).

### 3.2 Demographics

In the current study, 31 melasma patients completed the follow‐up. The demographic and clinical characteristics of the patients are shown in Table 1. The mean (SD) age of patients was 35.51 (8.25) years, with a range from 20 to 57 years. The duration of melasma was 3.29 (1.68) years, and most patients did not have a family history of this disease (28 patients; 90.3%) (Table 1). The most common risk factors that patients were exposed to were sun exposure (20 patients; 64.5%), followed by OCP use (8 patients; 25.8%). The type of melasma was classified as epidermal in 8 patients (25.8%), dermal in 4 patients (12.9%), and a combination of both dermal and epidermal in 19 patients (61.3%) (Table 1).

**TABLE 1:** Demographic and clinical characteristics of 31 patients with melasma.

Characteristics	Mean (SD)
Age (years)	35.52 (8.25)
Disease duration (years)	3.29 (1.68)
	**Frequency (%)**
**Family history**	
No	28 (90.3%)
Yes	3 (9.7%)
**Risk factors**	
Sun exposure	20 (64.5%)
OCP use	8 (25.5%)
Pregnancy	3 (9.7%)
**Type of melisma**	
Epidermal	8 (25.5%)
Dermal	4 (12.9%)
Mixed	19 (61.3%)

### 3.3 MASI Score

**FIGURE 1:**
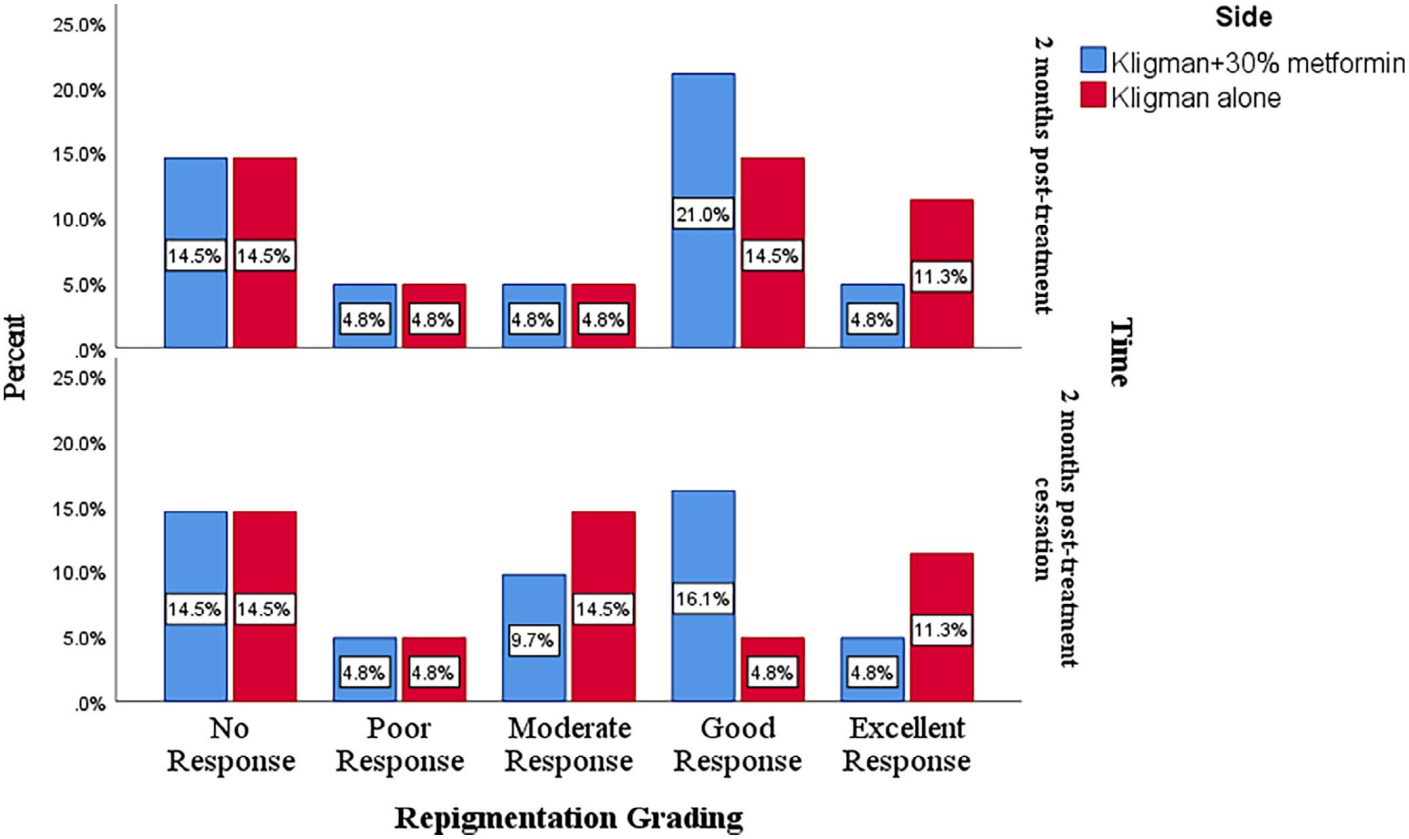
Changes in the MASI score on both treatment sides during study follow‐up.

As shown in Table 2 and Figure 1, a significant reduction was observed in the MASI score on both sides of the face at 2 months after treatment and 2 months after treatment cessation (*p*_for time_ < 0.001). The time × side interaction effect was statistically significant (*p* < 0.001), indicating that the reduction in the MASI score on the Kligman‐alone‐treated side was sharper than on the Kligman + 30% metformin‐treated side (Figure 1). The MASI score showed a higher reduction on the Kligman‐alone‐treated side over time from baseline, as the mean MASI score in the side that received Kligman‐alone was lower than the side that received Kligman + 30% metformin at 2 months after treatment (4.83 ± 0.91 vs. 5.90 ± 1.08; *p* = 0.096) and also 2 months after treatment cessation (4.94 ± 0.89 vs. 5.95 ± 1.07; *p* = 0.100). However, these differences were not statistically significant (*p* > 0.05) (Table 2). Two months after treatment, the mean MASI score significantly decreased from baseline on both sides: the Kligman + 30% metformin‐treated side (5.90 ± 1.08 vs. 9.06 ± 1.70; *p* = 0.004) and the Kligman‐alone‐treated side (4.83 ± 0.91 vs. 9.05 ± 1.71; *p* = 0.001). Although there was a slight increase in the mean MASI score 2 months after stopping treatment (4 months after treatment initiation) compared to 2 months after treatment on both sides, the scores remained significantly lower than baseline on the Kligman + 30% metformin‐treated side (5.95 ± 1.07 vs. 9.06 ± 1.70; *p* = 0.005) and on the Kligman‐alone‐treated side (4.94 ± 0.89 vs. 9.05 ± 1.71; *p* = 0.001) (Table 2).

**TABLE 2:** Comparison of changes in MASI score and VAS score over time based on the type of treatment received.

Time	Face (*n* = 31)		Between sides *p*‐value^a^	GEE *p*‐value	
	Kligman + 30% metformin treated side	Kligman alone treated side			
**MASI score**					
Baseline	9.06 ± 1.70	9.05 ± 1.71	> 0.999	Time	0.008*
2 months post‐treatment (2 months)	5.90 ± 1.08	4.83 ± 0.91	0.096	Side	< 0.001*
2 months post‐treatment cessation (4 months)	5.95 ± 1.07	4.94 ± 0.89	0.100	Time × Side	< 0.001*
Within side *p*‐value^a^	< 0.001*	< 0.001*			
Pairwise comparisons over time *p*‐value	Baseline vs. 2 months: 0.004* Baseline vs. 4 months: 0.005* 2 months vs. 4 months: > 0.999	Baseline vs. 2 months: 0.001* Baseline vs. 4 months: 0.001* 2 months vs. 4 months: > 0.999			
**VAS score**					
2 months post‐treatment (2 months)	5.29 ± 0.62	5.84 ± 0.68	0.422	Side	0.283
				Time	0.114
2 months post‐treatment cessation (4 months)	5.87 ± 0.53	6.23 ± 0.65	0.297	Time × Side	0.774
Within side *p*‐value^a^	0.196	0.402			

*Note:* The data are shown as Mean ± SE. *p*‐values are from a GEE with two within factors (time, side).

Abbreviation: GEE, Generalized Estimating Equation model.

^a^ Pairwise comparisons were done by Bonferroni adjustment for multiple comparisons.

* A *p*‐value < 0.05 was considered statistically significant.

### 3.4 Repigmentation Grading

**FIGURE 2:**
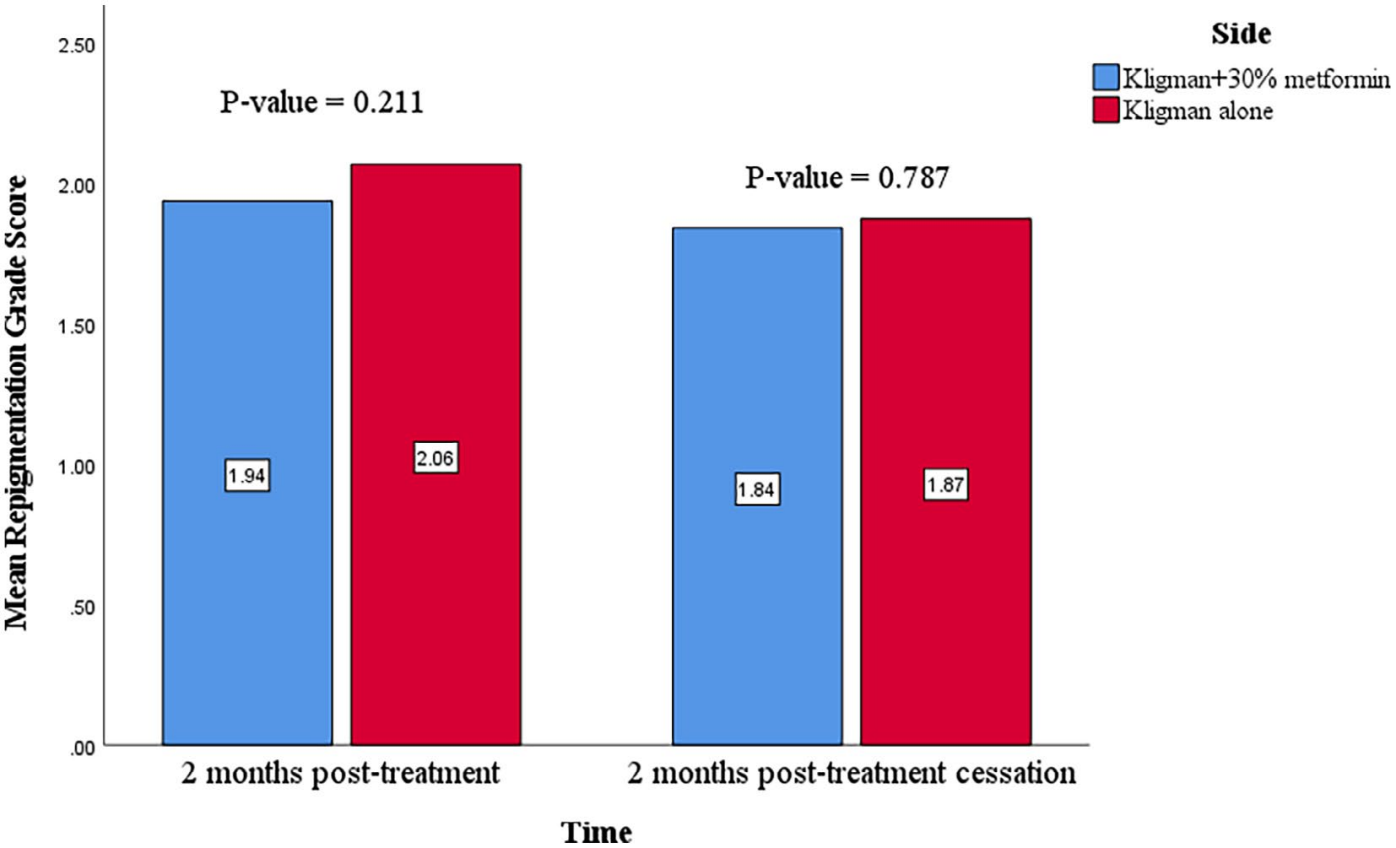
Comparison of mean repigmentation grade scores between treatment sides in a split‐face design at 2 months post‐treatment and 2 months after treatment cessation. Pairwise comparisons done with Bonferroni adjustment for multiple comparisons. No statistically significant differences were observed between the two sides at either time point.

The distribution of repigmentation grade responses between the two treated sides at two time points after treatment is shown in Figure 2. At 2 months post‐treatment, the side treated with Kligman + 30% metformin achieved a higher rate of good response (21.0%) compared to the Kligman‐only treated side (14.5%). In contrast, the Kligman‐only treated side showed a higher rate of excellent response (11.3% vs. 4.8%). Both sides exhibited equal proportions of no response (14.5%) and poor response (4.8%). At 2 months after stopping treatment, the Kligman + 30% metformin treated side retained more cases with a good response (16.1% vs. 4.8%), while the Kligman alone treated side showed higher rates of moderate (14.5%) and excellent response (11.3%) compared to the combination side. The proportions of no response (14.5%) and poor response (4.8%) remained similar between the two sides. Adding 30% metformin increased the chances of achieving a good response during active treatment and maintaining it after stopping, while Kligman's alone resulted in a higher proportion of excellent responses over the long term.

**FIGURE 3:**
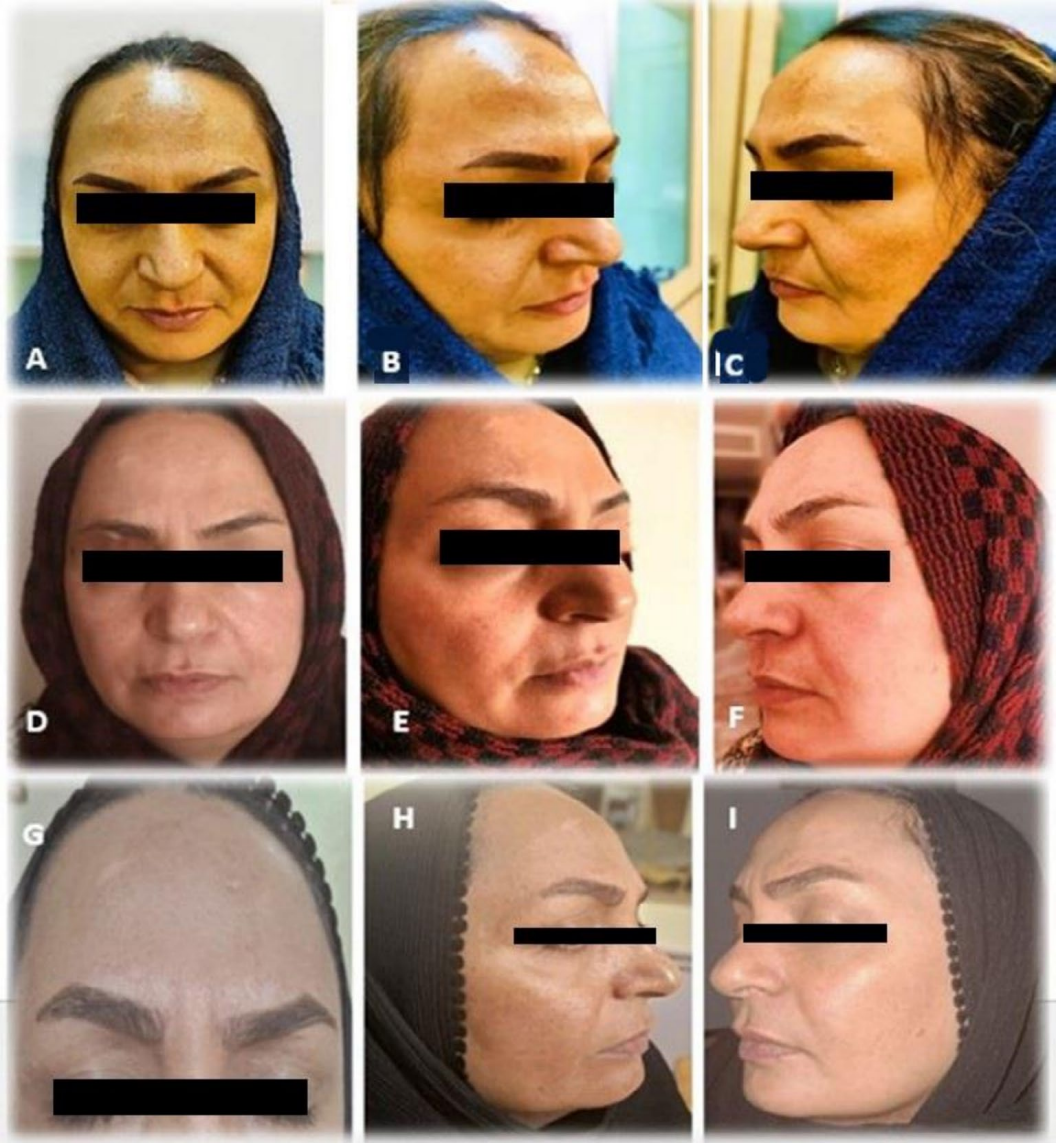
Clinical evaluation of melasma treatment response in one of the patients. (A) Frontal view before treatment. (B) Right facial side before treatment. (C) The left facial side before treatment. (D) Frontal view (2 months after treatment). (E) The right facial side was treated with Kligman's formula alone (2 months after treatment). (F) The left facial side was treated with a combination of Kligman's formula and topical metformin (2 months after treatment). (G) Frontal view 2 months after stopping treatment. (H) The right facial side 2 months after stopping treatment. (I) The left facial side 2 months after stopping treatment.

The mean repigmentaion score was slightly higher on the Kligman alone side compared with the Kligman + 30% metformin side at 2 months post‐treatment (2.07 ± 0.29 vs. 1.94 ± 0.26; *p* = 0.211), and at 2 months after treatment cessation (1.87 ± 0.27 vs. 1.84 ± 0.25; *p* = 0.787), though this difference was not statistically significant (Figure 3). The side treated with Kligman's formula alone showed a significant decrease in repigmentation grade scores after stopping treatment compared to the 2 months after treatment (1.87 ± 0.27 vs. 2.07 ± 0.29; *p* = 0.012), indicating a less lasting effect. In contrast, the Kligman + 30% metformin treated side did not display a significant decline (1.84 ± 0.25 vs. 1.94 ± 0.26; *p* = 0.083), implying that adding metformin may help sustain repigmentation results over time.

### 3.5 Patient's Satisfaction Score (VAS Score)

The patients' satisfaction scores measured by the VAS score were compared between and within the two treated sides over time. As shown in Table 2, there was no significant difference in the VAS score between the Kligman + 30% metformin‐treated side and the Kligman‐alone‐treated side at 2 months after treatment (5.29 ± 0.62 vs. 5.84 ± 0.68; *p* = 0.422), and also at 2 months after treatment cessation (5.87 ± 0.53 vs. 6.23 ± 0.65; *p* = 0.297). In both treated sides, 2 months after treatment cessation, the satisfaction score increased compared to 2 months after treatment; however, this increase was not significant (*p* > 0.05) (Table 2).

### 3.6 Side Effects

Results of side effect comparisons between the two treated sides showed that burning sensation was significantly more common in the side treated with Kligman alone than in the side treated with Kligman + 30% metformin (41.9% vs. 19.4%; *p* = 0.016). Meanwhile, inflammation (38.7% vs. 19.4%; *p* = 0.031) and redness (41.9% vs. 9.7%; *p* = 0.002) were more frequent in the Kligman + 30% metformin‐treated side compared to the Kligman‐alone treated side (Table 3). There was no significant difference in the frequency of irritation and hyperpigmentation between the two sides (*p* > 0.05) (Table 3).

**TABLE 3:** Comparisons of side effects between two treated sides.

Side effect	Face (*n* = 31)		*p*
	Kligman + 30% metformin treated side	Kligman alone treated side	
Irritation	9 (29%)	9 (29%)	> 0.999
Inflammation	12 (38.7%)	6 (19.4%)	0.031*
Hyperpigmentation	6 (19.4%)	9 (29%)	0.250
Burning sensation	6 (19.4%)	13 (41.9%)	0.016
Redness	13 (41.9%)	3 (9.7%)	0.002*

*Note:* The data are shown as frequency (%). *p*‐values are from a McNemar's test.

* A *p*‐value < 0.05 was considered statistically significant.

The results of the treatment can be seen in Figure 3.

## 4 Discussion

Melasma still remains a therapeutic challenge considering its chronicity and also the multifactorial pathogenesis, and high recurrence rate. Recent literature has been more focused on refining topical treatments by incorporating adjunctive agents in order to target multiple pathogenic pathways at the same time.

A study by Torok et al. evaluated the efficacy of various topical modalities. It showed hydroquinone as the most effective single agent. This study also highlighted the clinical benefit of combination treatments [17]. Supporting this, Iraji et al. [10, 18, 19, 20], conducted multiple studies demonstrating: (1) zinc sulfate as a safer alternative to hydroquinone with comparable efficacy; (2) enhanced pigmentation reduction with glycolic acid and hydroquinone combinations; and (3) significant clinical improvement with mesotherapy incorporating tranexamic acid, ascorbic acid, and glutathione.

In this context, metformin, which is actually a widely used antidiabetic agent, has attracted attention for its antimelanogenic, anti‐inflammatory, and antioxidative properties. Metformin inhibits melanogenesis through AMPK activation, MITF suppression, and downregulation of tyrosinase and related enzymes. Therefore, it can reduce melanin synthesis. Preclinical evidence has also demonstrated that metformin modulates the mTOR pathway and decreases oxidative stress, both of which contribute to pigment stabilization [20, 21].

In a randomized controlled trial, AboAlsoud et al. [17] found that a 30% metformin cream was shown to be effective compared to the triple combination cream (Kligman's formula), with fewer adverse events. This suggests its potential as a safer and tolerable alternative.

Our split‐face randomized trial compared Kligman's formula alone with a metformin‐containing combination. Both regimens resulted in significant reductions in pigmentation, with high patient satisfaction scores. Although Kligman's formula alone was associated with a slightly greater reduction in pigmentation darkness at the end of treatment, the addition of topical metformin showed two clinically meaningful advantages: (1) greater stability of repigmentation during follow‐up, and (2) absence of PIH, which occurred only in the Kligman‐only group. These findings suggest that metformin may enhance long‐term pigment control and reduce inflammatory reactions, potentially by decreasing cutaneous inflammation and oxidative stress.

Our results are consistent with previous clinical investigations. AboAlsoud et al. [17] reported that 30% topical metformin achieved efficacy comparable to the triple combination cream; however, with a better safety profile and fewer adverse events. Similarly, Ravikumar et al. [16] demonstrated the efficacy and tolerability of topical metformin in melasma patients, supporting its role as a safe adjunct.

While mild erythema was observed more frequently in the metformin group in our study, these reactions were transient and self‐limiting and did not lead to discontinuation. Additionally, the protective effect against PIH may make metformin particularly valuable for patients with darker skin types, who are at greater risk of pigmentary complications.

The high patient satisfaction observed across both groups emphasizes the acceptability of topical regimens, though long‐term adherence remains a challenge in chronic conditions like melasma. Our findings highlight metformin's potential as a maintenance strategy to sustain pigmentation improvement after initial therapy.

Nonetheless, several limitations should be acknowledged. The small sample size and relatively short follow‐up period may have a negative impact on the results. Although dermoscopy has been shown to improve morphological characterization of melasma, it was not available at our center during the study period. Future studies should incorporate dermoscopic or objective imaging tools to enhance lesion characterization. On the other hand, in our study, the histological or molecular assessments of melanogenesis markers were not included. This could have provided mechanistic insight. Future larger randomized trials with extended follow‐up and biomarker analysis are essential to validate these findings and define standardized protocols for melasma treatment algorithms.

## 5 Conclusion

In conclusion, the integration of metformin into a standard triple therapy for melasma is consistent with evolving therapeutic strategies aimed at maximizing efficacy while minimizing side effects. Our study supports the inclusion of topical metformin as a viable adjunct, particularly in patients prone to PIH or seeking enhanced pigment stability. Further large‐scale, multi‐center randomized trials with extended follow‐up and histologic correlation are warranted to substantiate these findings and establish standardized treatment protocols for this population.

## Authors' Contribution

F.I. designed the study. Z.K. and S.S. collected the data, conducted a literature review and drafted the manuscript. J.V. helped with the preparation of the therapeutic combination used. F.I and F.M supervised the process and helped with the analysis. S.S. analysed the collected data. All authors proofread and approved the final version of the manuscript and consented to be accountable for all aspects of the work.

## Funding

The authors have nothing to report.

## Ethics Statement

This study was approved by the Ethics Committee of Isfahan University of Medical Sciences (Code IR.MUI.MED.REC.1402.336) and by the registry for clinical trials. The study was conducted in compliance with the relevant guidelines and regulations, as well as the Declaration of Helsinki.

## Consent

Informed consent was signed by all the participants regarding participating in the study and data and photo sharing. They were assured that all the data stay anonymous. All participants consented for data and photo sharing. Written informed consent was obtained from all patients participating in this study after providing comprehensive information about the disease, available treatments, and the potential effects and complications of the current treatment. Patients were free to withdraw from the study at any time. Also, the confidentiality of patients' information was assured by the researcher.

## Conflicts of Interest

The authors declare no conflicts of interest.

## Data Availability

The findings of the current study are available upon request from the corresponding author.
